# Association between inflammatory markers, body composition and frailty in home-dwelling elderly: an 8-year follow-up study

**DOI:** 10.1007/s11357-024-01279-w

**Published:** 2024-07-09

**Authors:** Pia Bålsrud, Stine M. Ulven, Inger Ottestad, Kjetil Retterstøl, Ursula Schwab, Kirsten B. Holven

**Affiliations:** 1 Department of Nutrition, Institute of Basic Medical Sciences, University of Oslo, Oslo, Norway; 2 Clinical Nutrition, Department of Clinical Service, Division of Cancer Medicine, Oslo University Hospital, Oslo, Norway; 3 The Lipid Clinic, Oslo University Hospital, Oslo, Norway; 4 Institute of Public Health and Clinical Nutrition, University of Eastern Finland, Kuopio, Finland; 5 Department of Medicine, Endocrinology and Clinical Nutrition, Kuopio University Hospital, Wellbeing Services County of North Savo, Kuopio, Finland; 6 National Advisory Unit On Familial Hypercholesterolemia, Oslo University Hospital, Oslo, Norway

**Keywords:** Inflammageing, Longitudinal study, Older subjects, Frailty, Frailty Index, Ageing, Home-dwelling, Body composition

## Abstract

Frailty has been linked to inflammation and changes in body composition, but the findings are inconsistent. To explore this, we used the Frailty Index (FI) definition to (1) investigate the association between levels of inflammatory markers (baseline) and change in FI score after 8 years of follow-up and (2) investigate the longitudinal associations between inflammatory markers, body composition, and frailty. Home-dwelling elderly (≥ 70 years) were invited to participate in the study and re-invited to a follow-up visit 8 years later. This study includes a total of 133 participants. The inflammatory markers included were high-sensitive C-reactive protein (hs-CRP), interleukin 6 (IL-6), tumor necrosis factor alpha (TNF-α), and glycoprotein acetyls (Gp-acetyls). We used the body composition markers fat mass, fat-free mass, and waist circumference. The FI score consisted of 38 variables. Additional clinical assessments such as blood pressure and body mass index (BMI), as well as information about daily medications, were collected at both visits. Linear regression model and Spearman’s rank correlation were used to investigate associations. We showed that the FI score increased after 8 years, and participants with higher hs-CRP levels at baseline had the largest change in the FI score. Changes in fat mass were significantly correlated with changes in hs-CRP and IL-6, and changes in waist circumference were significantly correlated with changes in TNF-α. The use of drugs increased during the 8 years of follow-up, which may have attenuated the associations between inflammation and frailty. However, elevated concentrations of hs-CRP in the elderly may be associated with an increased risk of frailty in subsequent years.

**Supplementary Information:**

The online version contains supplementary material available at 10.1007/s11357-024-01279-w.

## Introduction

The global population is getting older, which substantiates the importance of healthy ageing, both for the individual itself and for the costs of society [1]. The goal is to increase the healthy active years, not only increase years of life [2].

Frailty increases the vulnerability to adverse outcomes and is associated with ageing [3, 4]. Frailty affects multiple domains: from physical function to mental and cognitive functioning [3]. The frailty condition is most commonly described by either the frailty phenotype by Fried et al. [5] or by the accumulation of deficits (Frailty Index, FI) by Rockwood et al. [6]. The biological mechanism of frailty is not fully understood; however, systemic inflammation has been suggested as a potential underlying mechanism of frailty [7, 8].

“Inflammageing,” a low-grade chronic inflammation during ageing, is commonly observed among older individuals [9]. Many mechanisms may contribute to inflammageing, such as cellular senescence, genetic susceptibility, central obesity, and microbiota composition, as well as direct inflammation-related mechanisms such as oxidative stress, dysregulation of inflammatory cells, and chronic infections [9]. The inflammatory state could further facilitate the development of cardiovascular disease, cardiovascular morbidity, and frailty [9, 10]. However, longitudinal studies are needed to investigate whether inflammation is a biomarker for biological ageing or if it is a cause of the pathology [9].

Ageing is associated with a decrease in skeletal muscle mass and an increase in fat mass [11]. Obesity and high waist circumference are shown to be associated with frailty phenotype, as well as a pro-inflammatory state [9, 11]. The age-related alterations in adipose tissue composition, increase in visceral adipose tissue, and decline in sex hormone levels promote low-grade chronic inflammation, and increase the risk of age-related diseases [12]. Weight loss, either by changes in lifestyle or unintentionally, has been shown to decrease the levels of inflammatory markers, and in combination with exercise, reduce some of the features of frailty in obese subjects [9].

Different inflammatory markers have been proposed as possible mediators of the development of frailty, where the most studied and positively associated with frailty are interleukin 6 (IL-6), high-sensitive c-reactive protein (hs-CRP), and tumor necrosis factor alfa (TNF-α) [8, 13, 14]. The FI has shown to be significantly positively associated with IL-6, TNF-α, and hs-CRP [15, 16], which means that higher levels of inflammatory markers are expressed in frail subjects. IL-6 is a commonly used marker of chronic inflammation and has properties such as inhibition of TNF-α and promote the production of CRP [8, 17]. CRP is an acute-phase protein, which eliminates pathogens and damaged cells when activated [14]. TNF-α is a cytokine, activated during pathological conditions and contributes to the activation of multiple metabolic pathways [14, 18].

The Gp-acetyl test is used as a clinical marker of systemic inflammation and is shown to increase with advancing age [19, 20]. The test measures changes in the N-glycan chains attached to acute-phase reactant proteins [21, 22]. Previous cross-sectional studies have shown a significant positive association between Gp-acetyls and frailty [23, 24] of which one used the FI definition.

The heterogeneity in the use of frailty definition, together with a mixture of cross-sectional and longitudinal results, contributes to inconsistent findings of the relationship between frailty and inflammatory markers. Thus, the aim of this study was to explore if inflammatory markers are associated with the development of frailty by investigating the association between (1) inflammatory markers at baseline and after follow-up and changes in FI after 8-year follow-up, (2) change in inflammatory markers and change in body composition after 8-year follow-up, and (3) change in body composition and change in FI after 8-year follow-up.

## Method

### Study subjects and design

The present study had a longitudinal study design. Home-dwelling people aged ≥ 70 years living in South-East Norway were recruited through the National Register in 2014/2015, where 2820 were invited to participate in the study [25]*.* In 2021, we did an updated extract from the National register. In 2022, participants who were still home-dwelling were invited to a new study visit, and 147 subjects participated in the follow-up study. In total, 133 subjects had sufficient data to calculate the FI score as previously described [26] and were included in this study (Supplementary Fig. 1)*.* The participants who came back for the follow-up visit were younger and healthier than the participants who did not come back for the follow-up visit. The participants met in a non-fasting state at both study visits, with no restrictions on dietary or lifestyle habits the day before the study visits. The baseline visit was conducted at Oslo and Akershus University College of Applied Science (changed name to Oslo Metropolitan University in 2018), and the follow-up visit was conducted at The University of Oslo (Department of Nutrition, UiO). To increase the participation rate, we also offered a follow-up visit at Oslo Metropolitan University, Kjeller, for participants who were not willing to travel to UiO.

The study was approved by the Regional Committees for Medical and Health Research Ethics, Health Region Southeast, Norway (107,167/REC), and followed the guidelines in the Declaration of Helsinki. Each participant had to sign an informed consent form at both study visits. To contact the former participants from 2014, the Norwegian Tax Administration approved and carried out an updated extraction from the National Population Registry.

### Frailty Index (FI)

We have previously described the FI score used in this study [26]. Briefly, we used data available in our study to make a retrospective FI based on five criteria from a standard procedure [6]: The variables included in the index must increase with age, be associated with health, not saturate at a too early age, cover a range of systems (to represent a holistic picture), and lastly, it should be consistent across time points if used serially. Thirty-eight deficits were included in the FI, scored between 0 and 1, where 0 is least frail and 1 is most frail [6]. All deficits were summarized and divided by the number of deficits available [26]. The FI score was used both as a continuous variable and as a categorical variable based on cutoff values from previous studies (frail ≥ 0.25, non-frail < 0.25) [27–31].

#### Components of the FI

The deficits included in the FI have previously been described [26], and the sub-categories of the index are presented in Supplementary Table 1**.** Briefly, data on diseases and conditions were collected by interview and by self-reported medication lists collected at the visits. Hemoglobin level, height, and weight were measured at the study visit, and body mass index (BMI) was calculated. The standardized questionnaires, Mini Nutritional Assessment (MNA ®) form, Short Form-36 (SF-36, version 2) [32], Mini-Mental State Examination (MMSE) form [33], Short Physical Performance Battery (SPPB) [34], were used to collect data on general daily functioning, self-reported health including mood/state of mind, cognitive function, and functioning, respectively at both the study visits. Grip strength was measured by using a handgrip dynamometer (KE-MAP80K1; Kern Map, Ballingen Germany), as previously described [25]. Three measurements were conducted at each hand, where the best result from the grip strength test was used in the FI score.

### Inflammatory markers

Non-fasting blood samples were collected by trained bioengineers. The samples from the baseline visit and the follow-up visit were measured together at the same time, and the samples from both visits were frozen and stored at − 80 °C until analysis, and they had not been previously thawed before analysis. Serum levels of hs-CRP were analyzed at FÜRST Medical Laboratory (Oslo, Norway) at each visit. The plasma levels of TNF-α, interferon-gamma (IFN-γ), interleukin 1 beta (IL-1β), and interleukin 10 (IL-10), and serum levels of IL-6 were measured using an ELISA kit (R&D Systems Inc., Minneapolis, USA), at the University of Oslo, Norway, in accordance with the protocol provided. All samples were measured in duplicates. Gp-acetyls were measured using magnetic resonance (NMR) spectroscopy (Nightingale, Finland).

The levels of IFN-γ, IL-1β, and IL-10 were very low in our study population, where 58% (baseline) and 63% (follow-up) had an IFN-γ < 0.5 pg/mL (detection level), 93% (baseline) and 96% (follow-up) had an IL-1β < 0.1 pg/mL (detection level), and 90% (baseline) and 75% (follow-up) had an IL-10 < 0.8 pg/mL (detection level). Thus, these inflammatory markers were not included in the further analyses.

### Body composition

Body composition was measured by a Bioelectrical impedance analysis (BIA) tool at both baseline (Tanita 100 bioimpedance analyzer, BC-418; Tanita Corp, Tanita Europe BW Amsterdam, Netherlands) and follow-up (SECA mBCA515 analyzer, Seca GmbH & Co. KG., Hamburg, Germany (*n* 102), or TANITA Body CompositionAnalyzer, MC-980 MA-N plus III, Tanita Europe BW Amsterdam, Netherlands (*n* 18)). Before analysis, the participants were asked to go to the toilet (if necessary), be lightly dressed, and take off metallic and electronic devices. If the participant had a pacemaker or other electronic implant, he/she was omitted from the analysis (baseline: *n* 3, follow-up: *n* 12). The body composition was calculated by using an internal formula. Waist circumference was measured between the lower edge of the costal arch and the top of the iliac crest. Hips circumference was measured by pulling the graduated tape around the widest point of the hips and buttocks. Waist and hip circumference were measured once per visit and read to the nearest centimeter(cm). The waist circumference was used both as a separate variable and included in the BIA measurement. To evaluate the fat-free mass, we calculated the fat-free mass index (FFMI) in accordance with the European Society for Clinical Nutrition and Metabolism (ESPEN) Consensus Statement [35]. FFMI was calculated by the equation FFMI = fat-free mass in kg / (height in meters)^2^. Cutoffs for low FFMI were < 15 kg/m^2^ for women and < 17 kg/m^2^ for men [35].

### Statistical analysis

Data with a normal distribution were presented as mean and standard deviation (SD), while data with a non-normal distribution were presented as median (25, 75 percentiles). For the categorical variables, data were presented as frequencies (*N*) and proportions (%). With the aim to study changes between variables at baseline and follow-up, a paired *t*-test was used for continuous variables with normally distributed data, while the Wilcoxon signed-rank test was used for continuous variables with non-normal distributed data. For paired categorical data, we used McNemar’s test. To answer our research questions, we used a linear regression model using change in FI (FI score at follow-up minus FI score at baseline) as the dependent variable, and inflammatory marker at baseline as the independent variable. The linear regression models were both unadjusted and adjusted for age, smoking, sex, and fat mass (percent). In an additional analysis, we also included the use of statins in the adjusted model; however, this did not change our results. Thus, statins were not included in the final model. Hs-CRP was divided into quartiles, and a linear regression model was performed for each quartile (exposure). Spearman’s rank correlation coefficient was used to investigate the correlation between change in inflammatory markers (levels at follow-up minus levels at baseline) and change in FI score (FI score at follow-up minus FI score at baseline), the correlation between the change in body composition (fat mass *(%, kg)*, fat-free mass *(kg, FFMI)*, and waist circumference *(cm)* at follow-up minus baseline) and the change in inflammatory markers, the correlation between the change in body composition and the change in FI, as well as the correlation between inflammatory markers.

## Results

### Characteristics of the study population

Of the 133 participants, 64 (48%) were women, and 69 (52%) were men. The median age was 73 years (25, 75p: 71, 75 years) at baseline, and 80 years (25, 75p: 79, 83 years) at follow-up, Table 1.

**Table 1:** Descriptive data and body composition of the study population

	**Baseline (*N* = 133)**		**Follow-up (*N* = 133)**		***p*-value**
	***Mean/median***	***SD/(25p, 75p)***	***Mean/median***	***SD/(25p, 75p)***	
**Female***, n (%)*	64	48.1	64	48.1	
**Age,** *years*	73	(71, 75)	80	(79, 83)	
**FI score**	0.11	(0.07, 0.16)	0.20	(0.13, 0.28)	** < 0.001**
**Frail***, n (%)*	4	3.0	44	33.1	** < 0.001**
**Polypharmacy (≥ 5 drugs/day),** *n(%)*	14	10.5	47	35.3	** < 0.001**
**Body weight,** *kg*	75.8	12.6	74.1	13.1	** < 0.001**
**Weight loss > 3 kg in the last 3 months,** *n(%)*					0.15
*No*	118	88.7	109	82.0	
*1–3 kg*	14	10.5	12	9	
> *3 kg*	1	0.8	12	9	
**Height,** *cm*	170.1	9.1	168.9	9.1	** < 0.001**
**BMI,** *kg/m*^*2*^	26.1	3.5	25.9	3.8	0.16
**Fat mass,** %*	30.2	8.4	34.6	9.0	** < 0.001**
**Fat mass**, kg*	23.0	7.7	25.4	8.2	** < 0.001**
**Fat-free mass**, kg*	53.0	10.6	48.3	10.8	** < 0.001**
**FFMI,** *kg/m*^*2*^	18.1	2.1	16.7	2.4	** < 0.001**
**Waist,** *cm*	95.5	11.3	93.2	12.3	** < 0.001**
**Hips, ***cm*	103.3	7.2	102.2	7.6	**0.001**

Bold entries indicates a *p*-value < 0.05

*BMI*, body mass index; *FFMI*, fat-free mass index; *FI score*, Frailty Index score; *SD*, standard deviation. *p*-values: continuous normal distributed data were tested by paired *t*-test, and continuous non-normal distributed data were tested by Wilcoxon sign-rank test. McNemar’s test was used for categorical data. Statistically significant level: *p*-value < 0.05. Normal distributed data is presented as mean (SD); non-normal distributed data is presented as median (25, 75 percentile).*Number of participants included in the analyses Baseline (*n* 130), follow-up (*n* 120)

At the follow-up visit, the median FI score was higher (0.20 vs 0.11, *p* < 0.001), and more subjects were categorized as frail (33% vs 3%, *p* < 0.001) when compared to the baseline visit. More subjects used ≥ 5 drugs per day at follow-up compared to baseline (35% vs 11%, *p* < 0.001, Table 1). The total body weight, height, fat-free mass (kg and FFMI), and waist and hip circumference were significantly lower, while the fat mass in percent and fat mass in kg were significantly higher at follow-up compared to baseline (*p* ≤ 0.001, Table 1). BMI remained stable during the 8-year follow-up.

A comparison of baseline characteristics of the participants who participated versus those who did not participate in the follow-up visit is shown in Supplementary Table 2. The participants who did not participate in the follow-up were frailer at baseline (0.17 vs 0.11 in FI score, and 24% vs 3% were categorized as frail at the baseline visit). The age was slightly higher for the participants who did not come back to the follow-up visit, and more subjects had a low MMSE in the not participating group. Taken together, it was the fittest participants who participated in the follow-up visit.

### Inflammatory markers

The level of hs-CRP and Gp-acetyls decreased significantly, while the levels of TNF-α significantly increased from baseline to follow-up, Table 2. No significant changes in the other measured inflammatory markers were found after 8-year follow-up. We found a significant positive correlation between all the inflammatory markers, as shown in Supplementary Table 3.

**Table 2:** Inflammatory markers at baseline and follow-up

	**Baseline (*N* = 133)**		**Follow-up (*N* = 133)**		***p*-value**
	***Mean/median***	***SD/(25p, 75p)***	***Mean/median***	***SD/(25p, 75p)***	
Hs-CRP*, *mg/l*	1.5	(0.8, 2.9)	0.9	(0.5, 1.8)	** < 0.001**
**IL-6*,**** pg/mL*	2.0	(1.3, 2.9)	2.2	(1.5, 3.1)	0.11
**TNF-α***, pg/mL*	0.83	(0.72, 1.1)	0.92	(0.73, 1.1)	**0.001**
**Gp-acetyls,** *mmol/l*	0.86	0.11	0.84	0.10	**0.002**

Bold entries indicates a *p*-value < 0.05

*Hs-CRP*, high-sensitive c-reactive protein; *Gp-acetyls*, glycoprotein acetyls; *IL-6*, interleukin 6; *TNF-α*, tumor necrosis factor alfa. *p*-values: normal distributed data is tested by paired *t*-test, while non-normal distributed data is tested by Wilcoxon sign-rank test. Statistically significant level: *p*-value < 0.05. Normal distributed data is presented as mean (SD); non-normal distributed data is presented as median (25, 75p).*CRP < 50 mg/dL (*n* 132 at baseline, *n* 133 at follow-up)

### Inflammatory markers and change in FI

**Fig. 1:**
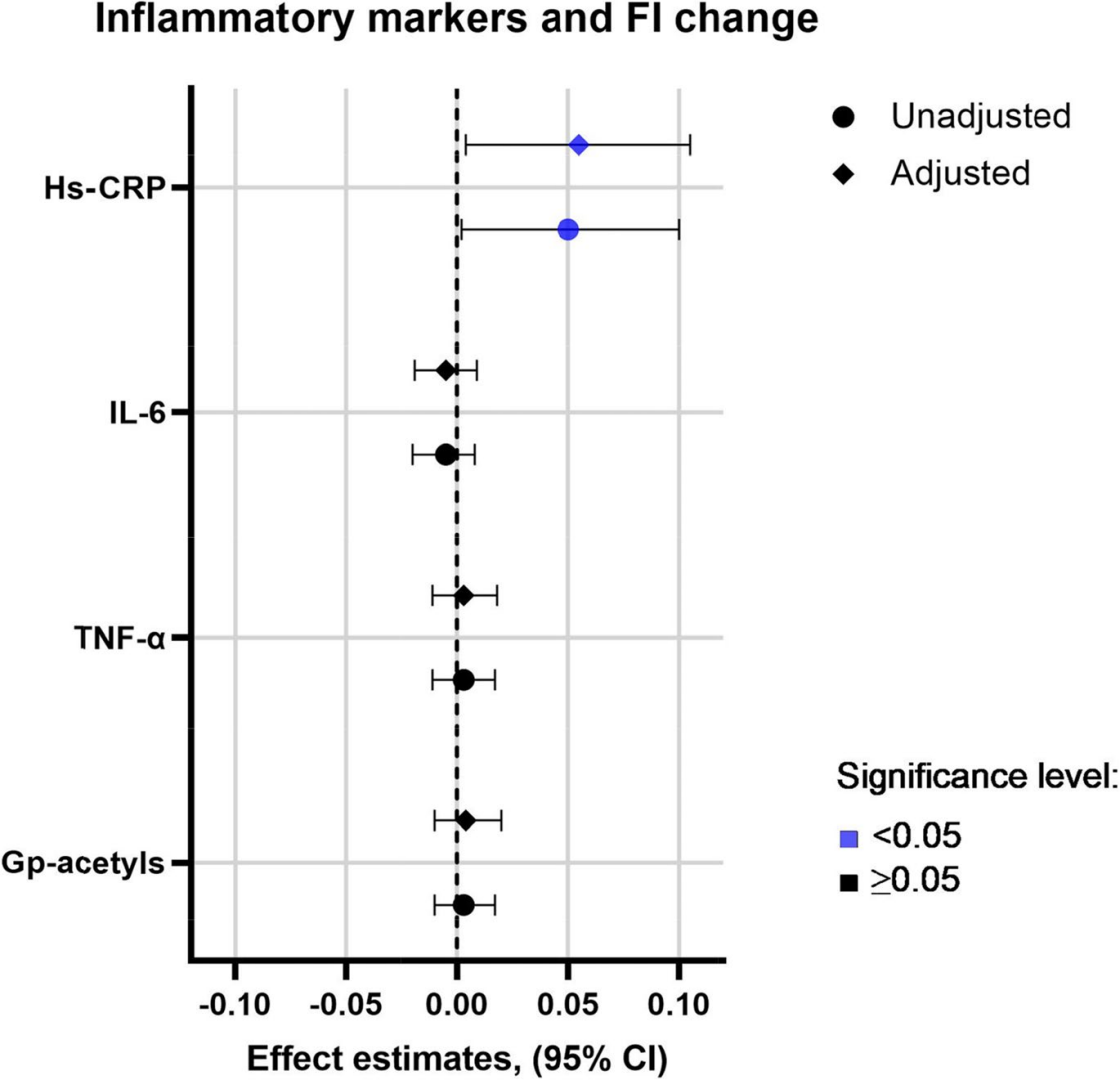
Forest plot of the results from the linear regression analyses between inflammatory marker at baseline (independent) and change in Frailty Index score (dependent) from baseline to follow-up. Due to the different scales of the inflammatory markers, we calculated the *z*-scores for all the variables to make them more presentable. The *z*-scores were calculated for each inflammatory marker by subtracting the mean and dividing by the standard deviation. The adjusted model was adjusted for age, smoking, sex, and fat mass (percent) at baseline. Hs-CRP, high-sensitive c-reactive protein; FI, Frailty Index; IL-6, interleukin 6; Gp-acetyls, glycoprotein acetyls; TNF-α, tumor necrosis factor alfa

We investigated if the levels of inflammatory markers at baseline could explain the change in FI score during the 8-year follow-up. The linear regression analysis showed a significant positive association between baseline hs-CRP and change in FI score both unadjusted and adjusted for age, sex, smoking, and fat mass in percent (Fig. 1, Table 3). In an additional analysis, we divided the concentrations of hs-CRP into quartiles and performed a linear regression model for each quartile, both unadjusted and adjusted. We showed that participants with the highest levels of hs-CRP at baseline showed a significant change in FI score after 8 years of follow-up and that these subjects were responsible for the association between hs-CRP and frailty ( Supplementary Table 4 and 5). No significant association between change in FI score and the other inflammatory markers was found. Further, we investigated the correlation between the change in inflammatory markers and the change in FI score, but no significant correlations were observed (data not shown).

**Table 3:** Linear regression analyses of the association between inflammatory markers and change in FI during the 8-year follow-up

Variables at baseline	Beta-coefficient	Model 1			Model 2			Model 3	
		**95% CI**	***p*-value**	**Beta-coefficient**	**95% CI**	***p*-value**	**Beta-coefficient**	**95% CI**	***p*-value**
Hs-CRP*, *mg/ml*	0.008	0.0004, 0.015	**0.04**	0.007	(0.0001, 0.15)	***0.05***	0.008	(0.0006, 0.015)	**0.03**
**IL-6***, pg/ml*	(− 0.002)	(− 0.008, 0.003)	0.41	(− 0.002)	(− 0.008, 0.004)	0.43	(− 0.002)	(− 0.008, 0.004)	0.51
**TNF-α***, pg/ml*	0.01	(− 0.04, 0.06)	0.67	0.01	(− 0.04, 0.06)	0.65	0.01	(− 0.04, 0.06)	0.66
**Gp-acetyls,** *mmol/l*	0.03	(− 0.10, 0.16)	0.64	0.03	(− 0.10, 0.16)	0.66	0.03	(− 0.10, 0.17)	0.62

Bold entries indicates a *p*-value < 0.05

*Hs-CRP*, high-sensitive c-reactive protein; *Gp-acetyls*, glycoprotein acetyls; *IL-6*, interleukin 6; *TNF-α*, tumor necrosis factor alfa. Model 1: inflammatory marker at baseline and change in FI score after 8-year follow-up (FI follow-up minus FI baseline) (*n* 133 for all, except CRP: *n* 132 (*n* 1 excluded due to hs-CRP > 50 mg/dl), Model 2: Model 1 adjusted for age, sex and smoking at baseline (*n* 133 for all, except hs-CRP: *n* = 132), Model 3: Model 1 adjusted for age, sex, smoking and fat mass (in percent) at baseline (for model 3 CRP: *n* 129, IL-6: *n* 130, TNF-α: *n* 130, due to unavailable BIA data). Statistically significant level: *p*-value < 0.05.*hs-CRP < 50 mg/dL

### Change in inflammatory markers and change in body composition

In the present study, we investigated the correlation between changes in markers of body composition (fat mass (%, kg), fat-free mass (kg, FFMI), and waist circumference (cm)) and changes in inflammatory markers. Spearman’s rank correlation analysis showed a significant positive correlation between change in fat mass (kg) and change in hs-CRP (rho 0.22, *p* = 0.01, Table 4), showing that a bigger change in fat mass was associated with a larger change in hs-CRP between the two visits. Changes in fat mass (%) fat mass (kg) and waist circumference were all significantly positively correlated to changes in IL-6 (rho 0.31, 0.32, 0.18, respectively, *p* < 0.05 for all). A change in waist circumference was significantly positively correlated with a change in TNF-α (rho 0.21, *p* = 0.02). No significant correlations were found for the other inflammatory markers, Table 4.

**Table 4:** Spearman’s rank correlation analysis between changes in inflammatory markers and changes in body composition markers

	**Fat mass (%)**		**Fat mass (kg)**		**Fat-free mass (kg)**		**Waist circumference (cm)**		**FFMI (kg/m**^**2**^**)**	
	**Spearman’s Rho**	***p*-value**	**Spearman’s Rho**	***p*-value**	**Spearman’s Rho**	***p*-value**	**Spearman’s Rho**	***p*-value**	**Spearman’s Rho**	***p*-value**
Hs-CRP_diff*	0.16	0.08	0.22	**0.01**	(− 0.04)	0.64	0.1	0.27	(− 0.07)	0.46
**IL-6_diff**	0.31	** < 0.001**	0.32	** < 0.01**	(− 0.08)	0.37	0.18	**0.04**	(− 0.12)	0.18
**TNF-α_diff**	0.08	0.39	0.13	0.16	0.05	0.56	0.21	**0.02**	0.004	0.96
**Gp-acetyls_diff**	0.13	0.16	0.13	0.16	(− 0.04)	0.65	0.15	0.10	(− 0.06)	0.49

Bold entries indicates a *p*-value < 0.05

*FFMI*; fat-free mass index; *hs-CRP_diff*, hs-CRP follow-up minus CRP baseline; *Gp-acetyls_diff*, Gp-acetyls follow-up minus Gp-acetyls baseline; *IL-6_diff*, IL-6 follow-up minus IL-6 baseline; *TNF-α*, TNF-α follow-up minus TNF-α baseline. *CRP < 50 mg/dL. Number of participants included in the analyses: hs-*CRP_diff*: at mass %, fat mass kg, and fat-free mass kg, FFMI (*n* 119), waist circumference (*n* 129). *IL-6 and TNF-α*: fat mass %, fat mass kg, and fat-free mass kg, FFMI (*n* 120), waist circumference (*n* 130). *Gp-acetyls*: fat mass %, fat mass kg, and fat-free mass kg, FFMI (*n* 119), waist circumference (*n* 129)

### Change in body composition and change in Frailty Index

There was no significant association between body composition markers at baseline and a change in FI score after 8 years (Supplementary Table 6). Nor did we find a significant correlation between changes in body composition and changes in FI score during the 8-year follow-up (data not shown).

### Sex-specific analyses

The hs-CRP levels decreased in both men and women during the 8-year follow-up (*p* < 0.001 for both, Supplementary Table 7**)**. The levels of TNF-α were significantly increased from baseline to follow-up for both men and women (*p* = 0.03 for both), while Gp-acetyls only decreased significantly in men (*p* = 0.007). No significant changes in IL-6 levels were observed between baseline and follow-up in either of the sexes, Supplementary Table 7.

The 8-year changes in body composition in men and women were the same as for the total study population, except for no significant change in waist and hip circumferences in men, Supplementary Table 8. Interestingly, women showed a larger increase in the categorization to “low FFMI” compared to men (W: from 8% at baseline to 60% at follow-up, vs M: 6% at baseline to 22% at follow-up, Supplementary Table 8).

## Discussion

In the present study, we found that subjects with high hs-CRP levels at baseline were more frail after 8 years. However, we did not find the same relationship for other inflammatory markers, nor for changes in inflammatory markers and changes in FI during the 8-year follow-up. The study population changed their body composition, showing a higher proportion of fat mass, and a lower proportion of fat-free mass at the follow-up visit compared to baseline. Further, this study showed a relationship between changes in both fat mass and waist circumference, and changes in inflammatory markers during the 8-year follow-up.

We have previously shown that IL-6, Gp-acetyls, and the inflammatory markers cathepsin S, cystatin C, and monocyte-specific gene expression were associated with FI [26]. Thus, in the present study, we wanted to investigate the longitudinal associations between inflammatory markers and changes in frailty and body composition during the 8-year follow-up.

Previous systematic reviews and meta-analyses have suggested that the inflammatory markers IL-6 and hs-CRP are markers of frailty [8, 13, 36]. However, these results are primarily based on Fried’s frailty phenotype definition [5] and are mainly findings from cross-sectional studies. Despite the significant findings in cross-sectional frailty phenotype data, Soysal et al. [36] did not find a significant association between IL-6 and hs-CRP, and frailty in the longitudinal studies included in their analyses.

High levels of TNF-α have also been significantly associated with frailty, but the results are not as consistent as for hs-CRP and IL-6 [8, 36]. Even though multiple studies have shown a significant association between frailty and the inflammatory markers hs-CRP, IL-6, and TNF-α, the literature is not unanimous [37].

Two studies from the systematic reviews and meta-analyses combined both the FI and the frailty phenotype definition and showed that higher levels of IL-6, hs-CRP, and TNF-α were significantly associated with increased risk of frailty when using both definitions [15, 16].

Longitudinal studies have also shown significant associations between FI and inflammatory markers. Welstead et al. [38] investigated the longitudinal associations between FI and hs-CRP. The participants were tested four times over 12 years and showed that higher levels of hs-CRP at baseline were associated with a worsening in FI score over 12 years. Hs-CRP was not associated with FI score in the cross-sectional analysis, or when using the frailty phenotype definition. Interestingly, these results are in line with our findings, showing no cross-sectional associations between FI and hs-CRP [26], but significant associations between high levels of hs-CRP at baseline and an increase in FI score change over time. Samson et al. [39] investigated trajectories of inflammatory markers over 20 years and showed that chronically elevated levels of IL-6 pathway markers (including hs-CRP) were associated with higher FI. They also suggested that the association may have been driven by overweight. Women showed stronger associations between inflammatory markers and frailty.

Our findings of an association between high levels of hs-CRP at baseline and more frailty after 8-year follow-up are in agreement with the studies previously described. However, in the present study, we found a decrease in the levels of hs-CRP in the follow-up period. This finding is most likely due to a large increase in the use of drugs between the two visits (≥ 5 drugs per day was 11% at baseline vs 35% at follow-up, *p* < 0.001). In particular, increased use of statin was observed leading to lower total and LDL cholesterol levels potentially impacting the inflammatory markers. In an additional analysis, we adjusted for the use of statins in the linear regression model. However, the adjustment did not change the results.

Previous studies have shown no significant differences in frailty between statin users and nonusers in neither women [40] nor in men [41]. However, a cross-sectional study on statin use and physical performance in patients on multiple drugs showed that statin users had better physical performance and muscle function compared to nonusers [42]. The effect of anti-inflammatory drugs on frailty progression (by the FI definition) has been investigated in subjects with cardiovascular disease (CVD), showing no significant effect [43]. Interestingly, a randomized controlled trial on aspirin use in men showed an inverse association between regular aspirin use and frailty (FI) [44]. In contrast, frequent use of non-aspirin nonsteroidal anti-inflammatory drugs (NSAIDs) was associated with higher frailty in the same study population [45].

The hs-CRP levels in our study population were low, compared to similar studies [15, 16, 38]. This may reflect a healthy population, also reflected by the low number of frail individuals at baseline (3%, Table 1). Even though there was a significant decrease in hs-CRP from baseline to follow-up, the levels were low at both time points.

The lack of significant associations between other inflammatory markers (IL-6, and TNF-α) and change in FI score may be due to low levels compared to similar studies [15, 16, 38, 39]. The inflammatory markers hs-CRP, IL-6, and TNF-α are closely associated and high baseline levels have previously been associated with increased mortality risk [46].

In agreement with our previous study [26], Mak et al. [23] showed that levels of Gp-acetyls were significantly associated with FI. No studies have studied the longitudinal association of Gp-acetyls to frailty [23]. Increased levels of Gp-acetyls have been associated with chronic inflammation, risk of CVD, and mortality [47]. In agreement with our findings, Gp-acetyls have previously been associated with the levels of hs-CRP, TNF-α, and IL-6 [48].

Previous studies have shown a positive association between overweight, obesity, and FI [49]. However, Hubbard et al. [50] showed a U-shaped curve between BMI and FI, where subjects with a BMI between 25–29.9 kg/m^2^ showed the lowest prevalence of frailty. In our study, BMI did not change during the study period.

Age-related changes in body composition, with an increased amount of visceral fat, can contribute to a higher inflammatory state [51]. Ageing of adipose tissue may affect other organs by infiltration of lipids, which further leads to systemic inflammation and acceleration of the ageing process [52]. In the previous study, we did find a correlation between changes in body composition and changes in inflammatory markers. Obesity has previously been described as a state of accelerated ageing, with inflammation and oxidative stress as common mediators [52, 53]. Interestingly, Khonsari et al. [54] investigated the association between normal-weight obesity (high-fat mass in percent) and inflammatory markers in their systematic review and meta-analysis and showed that normal-weight obesity was associated with high levels of hs-CRP and IL-6. In most of the studies included in this systematic review and meta-analysis, normal-weight obesity was defined as a fat percentage > 30% for women and > 25% for men. A normal BMI, but a high fat percentage, was also found in our study population both at baseline (for women only) and at follow-up (both for women and men, Supplementary Table 7).

Higher levels of hs-CRP and IL-6 have previously been shown to be associated with increased fat mass [55], as well as decreased muscle mass in older adults [56, 57]. Pedersen et al. [58] also showed an association between IL-6, TNF-α, and changes in fat distribution in older subjects. These findings agree with our findings of significant correlations between fat mass and IL-6; however, we did not find an association between fat mass and TNF-α. We found a significant change in FFMI from baseline to follow-up, where almost 50% of the women were categorized as having “low FFMI” at the follow-up visit. However, the change in FFMI was not significantly correlated with the change in inflammatory markers. We did find an association between changes in waist circumference and changes in IL-6 and TNF-α. These are interesting findings, as waist circumference is strongly related to visceral fat [59].

Gp-acetyls have previously been associated with BMI and are suggested to be elevated in line with adipose tissue-related low-grade inflammation [48]. We did not find any associations between body composition variables and Gp-acetyls in our study population. However, we did find an association with Gp-acetyls and all the other inflammatory markers, which underpins the systemic low-graded inflammatory state in older adults.

### Strengths and limitations

A strength of our study was the longitudinal design, following older subjects from their seventies to their eighties. This phase of life seems to be an important window of opportunities to promote healthy ageing and to prevent/delay frailty development. We used the FI to define frailty, which is interesting as most studies of inflammatory markers and frailty have used Fried’s frailty phenotype definition. Thus, our study is an important contribution to this research field. Our large available data material and use of standard procedures facilitated future research in the field of ageing.

Our study has also some limitations. The low participation rate at the follow-up visit (33.6% of the participants from the total number of participations at baseline) makes it reasonable to think that the frailest subjects did not participate at the follow-up visit thus limiting the generalizability of the study. We did not adjust for the use of anti-inflammatory drugs in our analyses, except for statins. We showed that more subjects used more than five drugs per day at follow-up compared to baseline and that the levels of hs-CRP were lower at follow-up compared to baseline. Thus, this might have affected our correlation analyses (weaker correlations than expected).

A major limitation of the study is that the BIA device used differed between the two study visits, and we cannot rule out that this may have impacted the results.

The COVID-19 pandemic may have had an impact on the present study; older and frailer subjects are more susceptible to severe COVID-19 [60], which may have affected our response rate. In addition to frailty and ageing, COVID-19 infection also affects the levels of inflammatory markers [60]. In Norway, all subjects aged > 65 years old were recommended to get vaccinated against COVID-19 [61] and the adherence to public vaccination programs is high. Thus, we can assume that most of our participants were vaccinated before they participated in this study. Lockdowns during COVID-19 and subsequent social isolation and decreased physical activity may have increased the risk of morbidity and frailty [62].

Finally, the plasma levels of the inflammatory markers measured were very low which may have impacted the possibility of picking out the correlation between inflammation and frailty.

## Conclusion

In the present study, we found that high levels of hs-CRP at baseline were significantly associated with a worsening in frailty in home-dwelling elderly during an 8-year follow-up. Also, the body composition changed during 8 years and was significantly associated with changes in inflammatory markers. There were no significant associations between other inflammatory markers and changes in FI score, nor any correlation between the changes in body composition and the changes in FI score. Our overall healthy study population together with the high medication consumption may have led to low levels of inflammatory markers; thus, the actual associations between inflammatory markers and frailty may be stronger than we have shown in this previous study.

## Supplementary Information

Below is the link to the electronic supplementary material.

Supplementary file1 (DOCX 115 KB)

## Data Availability

The data that support the findings of this study are not openly available due to reasons of sensitivity and are available from the corresponding author upon reasonable request. Data are located in controlled access data storage at University of Oslo.

## Abbreviations

BMI: Body mass index
CVD: Cardiovascular disease
hs-CRP: High-sensitive c-reactive protein
FFMI: Fat-free mass index
FI: Frailty Index
IL-6: Interleukin 6
MMSE: Mini Mental Status Evaluation
MNA: Mini Nutritional Assessment
SPPB: Short Physical Performance Battery
TNF-α: Tumor necrosis factor alfa
