# Supplementary material for: Association between inflammatory markers, body composition and frailty in home-dwelling elderly: an 8-year follow-up study
Source: GeroScience. 2024 Jul 9;46(6):5629–41. doi: 10.1007/s11357-024-01279-w (PMC11494618; doi:10.1007/s11357-024-01279-w)
Supplement: Supplementary file 1 — Supplementary file1 (DOCX 115 KB) [file 11357_2024_1279_MOESM1_ESM.docx]

**Supplementary Table 1:** The sub-categories of the FI score, and their contribution to the total frailty index.

| Sub-category of FI | Proportion of the total FI score (%) |
| --- | --- |
| Self-reported disease or condition | 26,3 |
| General daily function | 10,5 |
| Physical function and activity level | 21,1 |
| Self-reported health | 10,5 |
| Mood/State of mind | 21,1 |
| Cognitive function (MMSE) | 10,5 |

**Supplementary Table 2:**

|  | Participated in the follow-up  (N=133) | | Participated NOT in the follow-up (N=270) | | P-value |
| --- | --- | --- | --- | --- | --- |
|  | ***Mean/median*** | ***SD/(25p,75p)*** | ***Mean/median*** | ***SD/(25p,75p)*** |  |
| Female*, n (%)* | 64 | 48.1 | 146 | 54.1 | 0.26 |
| Age, *years* | 73 | (71, 75) | 76 | (73, 79) | <0.001 |
| BMI, *kg/m^2^* | 26.1 | 3.5 | 26.2 | 4.1 | 0.74 |
| FI score | 0.11 | (0.07, 0.16) | 0.17 | (0.11, 0.24) | <0.001 |
| Frail*, n (%)* | 4 | 3.0 | 66 | 24.4 | <0.001 |
| Single household*, n (%)* | 39 | 29.3 | 98 | 36.2 | 0.16 |
| MMSE score | 30 | (26, 30) | 27 | (25, 30) | <0.001 |
| *< 24 points,* n (%) | 0 | 0 | 22 | 8.2 | <0.001 |
| MNA score | 28.5 | (27.5, 29) | 27.5 | (26.5, 29) | <0.001 |
| SPPB score | 12 | (11, 12) | 11 | (10, 12) | <0.001 |
| *≤ 8 points, n (%)* | 0 |  | 22 | 8.2 | <0.001 |
| Grip strength (kg), *dominant* | 30.9 | (22.3, 40.4) | 26.1 | (19.4, 35.7) | <0.001 |
| Current smoker*, n (%)* | 5 | 3.8 | 19 | 7.0 | 0.19 |
| CVD*, n (%)* | 28 | 21.1 | 86 | 31.9 | 0.02 |
| Diabetes*, n (%)* | 7 | 5.3 | 12 | 4.4 | 0.72 |
| Polypharmacy  (≥5 drugs/day), *n (%)* | 14 | 10.5 | 47 | 17.4 | 0.7 |

*Normal distributed data; mean and SD, Non-normal distributed data; median (25p, 75p), categorical data; number and percent. For continuous variables: normal distributed data were tested by independent sample t-test, non-normal distributed data were tested by Wilcoxon rank-sum test. For categorical variables: Chi-square test or Fisher exact test (if the expected cell frequency was < 5).*

**Supplementary Table 3:** Spearman’s rank Correlation between inflammatory markers

| Inflammatory marker, baseline | Inflammatory marker, baseline | Spearman’s rho | P-value |
| --- | --- | --- | --- |
| Hs-CRP* | IL-6 | 0.38 | **<0.001** |
| Hs-CRP* | TNF-α | 0.27 | **0.002** |
| IL-6 | TNF-α | 0.42 | **<0.001** |
| Gp-acetyls | CRP* | 0.36 | **<0.001** |
| Gp-acetyls | TNF-a | 0.44 | **<0.001** |
| Gp-acetyls | IL-6 | 0.34 | **<0.001** |
|  |  |  |  |
| Change in inflammatory marker | **Change in inflammatory marker** | **Spearman’s rho** | **P-value** |
| Hs-CRP_diff* | IL-6_diff | 0.33 | **<0.001** |
| Hs-CRP_diff* | TNF-α_diff | 0.30 | **<0.001** |
| IL-6_diff | TNF-α_diff | 0.33 | **<0.001** |
| Gp-acetyls_diff | CRP_diff * | 0.26 | **0.002** |
| Gp-acetyls_diff | TNF- α _diff | 0.43 | **<0.001** |
| Gp-acetyls_diff | IL-6_diff | 0.40 | **<0.001** |

*Hs-CRP, high-sensitive c-reactive protein; Gp-acetyls, Glycoprotein Acetyls; IL-6, Interleukin 6; TNF-α, Tumor Necrosis Factor alfa. .” _diff” = (Level of inflammatory marker at follow-up minus the level of inflammatory marker at baseline). Statistically significant level: P-value <0.05. * hs-CRP<50 mg/dL*

**Supplementary Table 4:** Concentration of hs-CRP divided into quartiles

| Hs-CRP baseline | Mean | SD |
| --- | --- | --- |
| Hs-CRP, Q1 | 0.53 | 0.17 |
| Hs-CRP, Q2 | 1.2 | 0.21 |
| Hs-CRP, Q3 | 2.1 | 0.36 |
| Hs-CRP, Q4 | 4.6 | 2.1 |

*Hs-CRP, high-sensitive c-reactive protein. Unit used; mg/L.*

**Supplementary Table 5:** Linear regression model between quartiles of hs-CRP and change in FI score.

|  | Model 1 | | | Model 3 | | |
| --- | --- | --- | --- | --- | --- | --- |
| Hs-CRP baseline | **Beta-Coefficient** | **95% CI** | **P-value** | **Beta-Coefficient** | **95% CI** | **P-value** |
| Hs-CRP, Q1 | 0.06 | (-0.07, 0.19) | 0.34 | 0.04 | (-0.1, 0.18) | 0.61 |
| Hs-CRP, Q2 | 0.045 | (-0.1, 0.19) | 0.54 | 0.06 | (-0.1, 0.23) | 0.44 |
| Hs-CRP, Q3 | (-0.03) | (-0.1, 0.04) | 0.41 | (-0.02) | (-0.1, 0.06) | 0.55 |
| Hs-CRP, Q4 | 0.021 | (0.006, 0.04) | **0.009** | 0.02 | (0.007, 0.04) | **0.008** |

*Hs-CRP, high-sensitive c-reactive protein. Model 1: unadjusted. Model 3: adjusted for sex, age, smoking and fat mass (%) at baseline. Statistically significant level: P-value <0.05. CRP >50mg/L was excluded from the analysis (n=1)*

**Supplementary Table 6:** Linear regression model between body composition markers at baseline and change in FI score after 8 year follow-up.

|  | Model 1 | | | Model 3 | | |
| --- | --- | --- | --- | --- | --- | --- |
| Variables (baseline) | **Beta-coefficient** | **95% CI** | **P-value** | **Beta-coefficient** | **95% CI** | **P-value** |
| Body weight*, kg* | 0.00004 | (-0.001, 0.001) | 0.95 | 0.0003 | (-0.001, 0.002) | 0.62 |
| Fat mass, *%* | 0.0004 | (-0.001,0.002) | 0.60 | 0.0002 | (-0.002, 0.003) | 0.85 |
| Fat mass, *kg* | 0.0006 | (-0.001, 0.002) | 0.50 | 0.0006 | (-0.001, 0.003) | 0.58 |
| Fat-free mass, *kg* | (-0.0002) | (-0.002, 0.001) | 0.82 | 0.008 | (-0.002, 0.003) | 0.56 |
| Waist, *cm* | 0.0001 | (-0.001, 0.001) | 0.88 | 0.0003 | (-0.001, 0.002) | 0.67 |
| Hips, *cm* | 0.001 | (-0.0006, 0.003) | 0.16 | 0.001 | (-0.0006, 0.003) | 0.18 |

*Model 1: unadjusted. Model 3: adjusted for sex, age, smoking and fat mass (%) at baseline. Statistically significant level: P-value <0.05.*

**Supplementary Table 7:** The levels of inflammatory markers by sex

| Inflammatory marker | Baseline (N=133) | | | | | Follow-Up (N=133) | | | |  |  |
| --- | --- | --- | --- | --- | --- | --- | --- | --- | --- | --- | --- |
|  | | **Women (N=64)** | | **Men (N=69)** | | **Women (N=64)** | | **Men (N=69)** | | **P-value**† | **P-value**‡ |
|  | | ***Mean/median*** | ***SD/(25p,75p)*** | ***Mean/median*** | ***SD/(25p,75p)*** | ***Mean/median*** | ***SD/(25p,75p)*** | ***Mean/median*** | ***SD/(25p,75p)*** |  |  |
| Hs-CRP*, *mg/ml* | | 1.6 | (0.9, 2.9) | 1.3 | (0.7, 2.4) | 0.95 | (0.55, 1.95) | 0.9 | (0.5, 1.7) | **<0.001** | **<0.001** |
| IL-6*, pg/ml* | | 1.8 | (1.3, 2.6) | 2.1 | (1.5, 3.1) | 2.1 | (1.5, 3.1) | 2.3 | (1.6, 3.7) | ***0.06*** | 0.63 |
| TNF-α*, pg/ml* | | 0.82 | (0.69, 0.99) | 0.86 | (0.75, 1.12) | 0.89 | (0.71, 1.11) | 0.94 | (0.75, 1.10) | **0.03** | **0.03** |
| Gp-acetyls, *mmol/l* | | 0.88 | 0.11 | 0.85 | 0.10 | 0.86 | 0.10 | 0.82 | 0.10 | 0.10 | **0.007** |

*Hs-CRP, High-sensitive c-reactive protein; Gp-acetyls, Glycoprotein Acetyls; IL-6, Interleukin 6; TNF-α, Tumor Necrosis Factor Alfa. Normal distributed data is presented as mean (SD); non-normal distributed data is presented as median (25, 75p). Normal distributed data are tested by paired t-test, while non-normal distributed data are tested by Wilcoxon sign rank test. Statistically significant level: P-value <0.05. * hs-CRP< 50* *mg/dL (n 132 at baseline, n 133 at follow-up), P-value*†*: women baseline vs follow-up, P-valu*e‡: *men baseline vs follow-up.*

**Supplementary Table 8:** Body composition by sex

|  | Baseline (N=133) | | | | Follow-Up (N=133) | | | | P-value† | P-value‡ |
| --- | --- | --- | --- | --- | --- | --- | --- | --- | --- | --- |
|  | **Women (N=64)** | | **Men (N=69)** | | **Women (N=64)** | | **Men (N=69)** | |  |  |
|  | ***Mean/n*** | ***SD/(%)*** | ***Mean/n*** | ***SD/(%)*** | ***Mean/n*** | ***SD/(%)*** | ***Mean/n*** | ***SD/(%)*** |  |  |
| Body weight, *kg* | 69.0 | 10.4 | 82.1 | 11.2 | 67.3 | 11.3 | 80.3 | 11.6 | **0.001** | **<0.001** |
| Weight loss > 3 kg in the last 3 months, *n (%)* |  |  |  |  |  |  |  |  | 0.12 | 0.62 |
| *No* | 57 | (89.1) | 61 | (88.4) | 52 | (81.3) | 57 | (82.6) |  |  |
| *1-3 kg* | 7 | (10.9) | 7 | (10.1) | 7 | (10.9) | 5 | (7.3) |  |  |
| *>3 kg* | 0 |  | 1 | (1.5) | 5 | (7.8) | 7 | (10.1) |  |  |
| Height, *cm* | 162.6 | 5.1 | 177.0 | 6.0 | 161.4 | 5.0 | 175.8 | 5.9 | **<0.001** | **<0.001** |
| BMI, *kg/m^2^* | 26.0 | 3.7 | 26.2 | 3.3 | 25.8 | 4.1 | 26.0 | 3.5 | 0.33 | 0.31 |
| Fat mass, *%** | 36.4 | 5.4 | 24.5 | 6.5 | 41.0 | 6.7 | 28.2 | 6.1 | **<0.001** | **<0.001** |
| Fat mass, *kg** | 25.6 | 7.2 | 20.6 | 7.4 | 27.9 | 8.1 | 22.9 | 7.9 | **<0.001** | **<0.001** |
| Fat-free mass, *kg** | 43.6 | 4.3 | 61.5 | 6.5 | 39.1 | 5.1 | 56.7 | 6.7 | **<0.001** | **<0.001** |
| FFMI, *kg/m^2^** | 16.5 | 1.3 | 19.6 | 1.5 | 14.9 | 1.7 | 18.4 | 1.7 | **<0.001** | **<0.001** |
| Low FFMI, *n (%)** | 5 | 8.1 | 4 | 5.8 | 36 | 60.0 | 13 | 21.6 | **<0.001** | **0.01** |
| Waist, *cm* | 91.6 | 10.9 | 99.1 | 10.4 | 87.9 | 12.2 | 98.0 | 10.3 | **<0.001** | 0.07 |
| Hips, *cm* | 104.0 | 8.3 | 102.7 | 6.0 | 102.3 | 8.3 | 102.1 | 7.0 | **0.001** | 0.17 |

*SD, Standard deviation, FFMI, Fat-free mass index, Low FFMI is defined as <15* kg/m^2^ for women and < 17 kg/m^2^ for men*. Normal distributed data is presented as mean (SD), categorical variables are presented as frequencies and percent. P-values: Continuous normal distributed data were tested by paired t-test. McNemar’s test was used for categorical data. Statistically significant level: P-value <0.05. *Baseline: Women: n 62, Men: n 68; Follow-up: Women: n 60, Men: n 60;* P-value†*; between baseline and follow-up for women,* P-value‡*; between baseline and follow-up for men*

**Supplementary Figure 1:** Flow chart of the study population


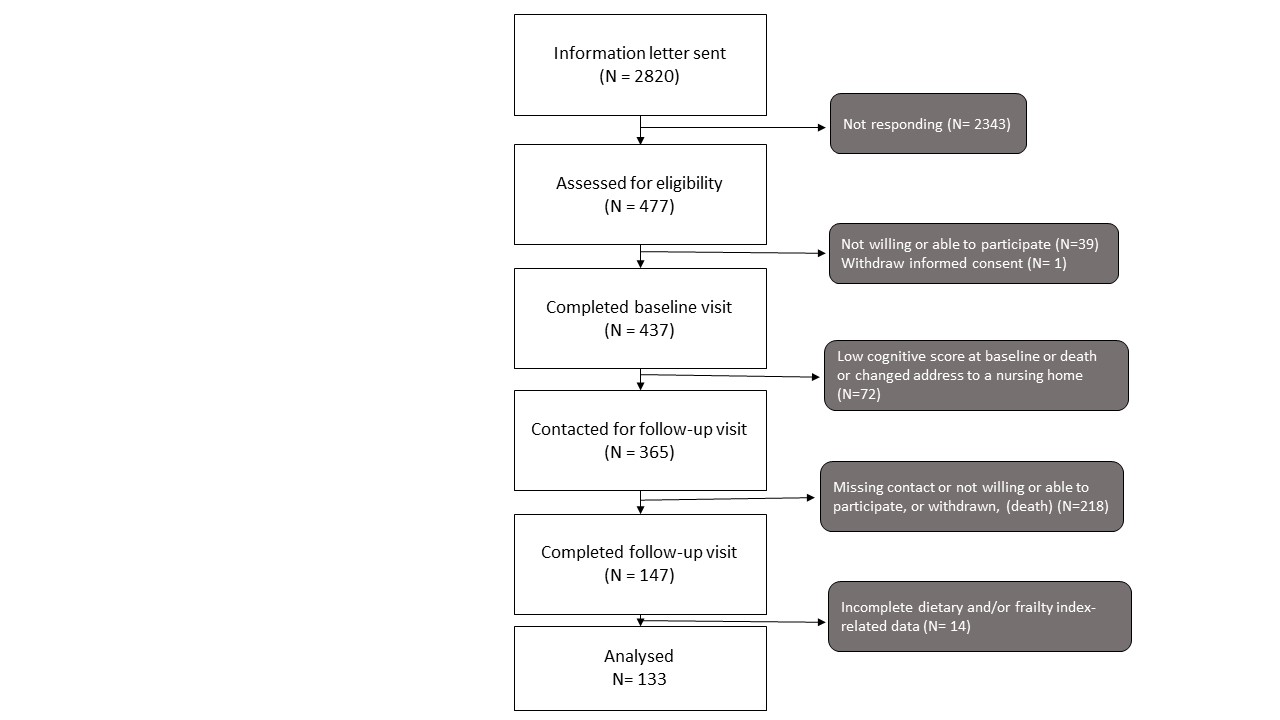


Figure text: The flow chart shows the flow from the participants invited to to the first study visit, to the number of participants included in this paper.
